# Abouchement séparé du canal pancréatique principale et du cholédoque: est- il synonyme de cholangiographie rétrograde par voie endoscopique facile?

**DOI:** 10.11604/pamj.2018.29.55.14597

**Published:** 2018-01-21

**Authors:** Mohammed Amine Benatta

**Affiliations:** 1 Unité d’Endoscopie Digestive, Hopital Central de l’Armée, Alger, Algérie

**Keywords:** Abouchement pancréatique et biliaire séparé, prothèse pancréatique, Separated pancreatic and biliary entrance, pancreatic stent

## Abstract

This study focused on endoscopic examination of a prominent papilla which showed two separate orifices revealing separate entrance of the main pancreatic duct and of the common bile duct (A). This was the most rare anatomoendoscopic variation, accounting for only 10% of retrograde cholangiopancreatographies using endoscopic approach (ERCP). However, it facilitated selective biliary catheterization (SBC) in our patient, as one might have expected. Papilla had a pseudo polypoid appearance and a biliary orifice (BO) at the right angle toward the duodenal wall; hence this was a more difficult approach than that via the pancreatic orifice (PO) which, on the other hand, required undesired pancreatic catheterization (B). As SBC was the approach we decided to adopt, the special techniques to consider in this particular case were: transpancreatic sphincterotomy (TPS) alone or TPS after implantation of a pancreatic stent (PS). We opted for the second technique (STP + PS) and we noticed that the implantation of a PS resulted in better BO visualization (C). We could perform SBC without recourse to TPS. In this specific case, as in other cases of difficult SBC in our practice, PS has proven effectiveness.

## Image en médecine

C'est une Papille proéminente dont l'examen endoscopique objective deux orifices distincts qui représente un abouchement séparé du canal pancréatique principale et du cholédoque (A). Il s'agit de la variante anatomoendoscopique la plus rare avec une fréquence de seulement 10% au cours des cholangiopancreatographies rétrogrades par voie endoscopique (CPRE). Pour autant cela allait il faciliter le cathétérisme biliaire sélectif (CBS) chez notre patient comme on aurait pu le supposé. La papille telle qu'elle se présente avec cet aspect pseudo polypoide et un orifice biliaire (OB) en position d'angle droit par rapport au mur duodénal était plus difficile d'acces au contraire de l'orifice pancréatique (OP) qui s'y prêtait plutôt facilement mais inévitablement a un cathétérisme pancréatique non désiré (B). Le CBS ayant été notre objectif les techniques spéciales a envisager devant ce cas précis étaient: soit la sphinctérotomie transpancréatique (STP) seule soit la STP après mise place d'une Prothèse Pancréatique (PP). Ayant opter pour la seconde technique STP + PP nous avons réaliser que des la mise en place de la PP cela a permis de mieux exposer l'OB (C). Le CBS a été finalement obtenu sans recourir a la STP. Dans ce cas précis comme dans d'autres cas de CBS difficile dans notre pratique, la PP a prouver son intérêt.

**Figure 1:**
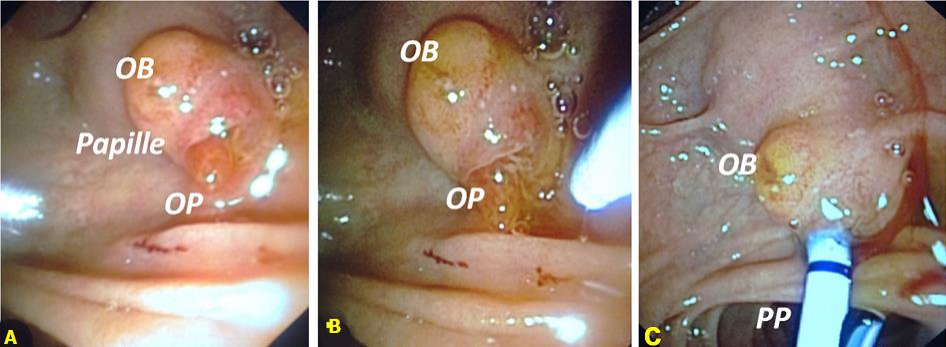
Prothèse pancréatique dans le cathétérisme biliaire sélectif en cas de papille avec abouchement séparé du canal pancréatique principale et du cholédoque

